# Proteome Analysis in a Mammalian Cell Line Reveals that PLK2 Is Involved in Avian Metapneumovirus Type C (aMPV/C)-Induced Apoptosis

**DOI:** 10.3390/v12040375

**Published:** 2020-03-28

**Authors:** Rong Quan, Li Wei, Lei Hou, Jing Wang, Shanshan Zhu, Zixuan Li, Moran Lv, Jue Liu

**Affiliations:** Beijing Key Laboratory for Prevention and Control of Infectious Diseases in Livestock and Poultry, Institute of Animal Husbandry and Veterinary Medicine, Beijing Academy of Agriculture and Forestry Sciences, No. 9 Shuguang Garden Middle Road, Haidian District, Beijing 100097, China; qrcau@126.com (R.Q.); w_lyx2008@126.com (L.W.); hlbj09@163.com (L.H.); jingjing_0047@sina.com (J.W.); x20632102@163.com (Z.L.);

**Keywords:** aMPV/C, differentially expressed proteins, iTRAQ, PLK2, apoptosis

## Abstract

Avian metapneumovirus subtype C (aMPV/C) causes an acute respiratory disease that has caused serious economic losses in the Chinese poultry industry. In the present study, we first explored the protein profile in aMPV/C-infected Vero cells using iTRAQ quantitative proteomics. A total of 921 of 7034 proteins were identified as significantly altered by aMPV/C infection. Three selected proteins were confirmed by Western blot analysis. Bioinformatics GO analysis revealed multiple signaling pathways involving cell cycle, endocytosis, and PI3K-Akt, mTOR, MAPK and p53 signaling pathways, which might participate in viral infection. In this analysis, we found that PLK2 expression was upregulated by aMPV/C infection and investigated whether it contributed to aMPV/C-mediated cellular dysfunction. Suppressing PLK2 attenuated aMPV/C-induced reactive oxygen species (ROS) production and p53-dependent apoptosis and reduced virus release. These results in a mammalian cell line suggest that high PLK2 expression correlates with aMPV/C-induced apoptosis and viral replication, providing new insight into the potential avian host cellular response to aMPV/C infection and antiviral targets.

## 1. Introduction

Avian metapneumovirus (aMPV) is the causative agent of respiratory tract infections and reduced egg production in poultry and has caused global economic losses [1,2]. aMPV was first reported in South Africa in 1978 [3] and was subsequently reported in many countries worldwide. Both aMPV and human metapneumovirus (hMPV) belong to the new *Pneumoviridae* family, genus *Metapneumovirus*. There are four subtypes of aMPV, termed A, B, C and D, which are based on genetic and antigenic differences [4]. There is a high similarity between subtype C aMPV and hMPV [5,6,7]. The infection of subtype C aMPV (aMPV/C) in Muscovy ducks has been reported in France and China [8,9] as well as in turkeys and wild birds in the USA [10], forming two distinct genetic lineages, one North American and the other Eurasian. In July 2010, an outbreak with severe respiratory symptoms occurred in meat chickens in southern China. Subsequently, we isolated a subtype C aMPV (aMPV/C), designed strain JC, from the infected chickens and showed that it was the causative agent. This was the first report of chickens infected with aMPV/C [11]. Subsequently, aMPV/C was reported to experimentally infect mice and induce strong inflammation in the lungs [12]. Another research study showed that aMPV/C could induce a complete autophagic response, which was partially regulated by ER stress [13]. However, analysis of proteomic profiles in aMPV/C-infected cells has not yet been described.

Currently, high-throughput proteomic approaches are effectively used for the comprehensive analysis of virus infection. Isobaric tags for relative and absolute quantification (iTRAQ) combined with liquid chromatography/tandem mass spectrometry (LC-MS/MS) analysis is a sensitive and accurate proteomic approach that has been widely employed to investigate proteomic profiles of host cells during viral infections, including highly pathogenic avian influenza (HPAI) virus [14], hepatitis B virus (HBV) [15], transmissible gastroenteritis virus (TEGV) [16], porcine circovirus type 2 (PCV2) [17], classical swine fever virus (CSFV) [18], porcine reproductive and respiratory syndrome virus (PRRSV) [19] and porcine epidemic diarrhea virus (PEDV) [20,21]. These studies have quantified cellular proteins and identified potential biomarkers in the infected cells, which also help improve our understanding of viral pathogenesis.

PLK2 (also called Snk), which belongs to a highly conserved mammalian serine/threonine protein kinase family, has an N-terminal serine/threonine kinase domain and a C-terminal polo-box domain (PBD). PLKs play multiple roles throughout the cell cycle and have emerged as anticancer therapeutic drug targets [22]. PLK2 was originally regarded as an immediate–early response gene and cell cycle regulator [23,24] and has been reported to display a complex functional response by acting as a cell survival factor [25,26,27] but occasionally as an apoptotic effector [28,29,30,31,32,33,34] in tumorigenesis and certain diseases. PLK2 signaling via p53 is involved in checkpoint signaling and tumor growth [31,35,36,37]. Studies have also revealed that the PLK2 response to oxidative stress is associated with p53 signaling [38,39].

To date, no study has been carried out to assess the protein profiles of aMPV/C-infected cells. In the present study, iTRAQ combined with LC-MS/MS was used to reveal the global proteomic changes occurring in Vero cells in response to aMPV/C infection for the first time and will help understand the molecular mechanisms underlying viral infection. We found that PLK2 expression was upregulated and further examined its biological function in aMPV/C infection. Suppression of PLK2 by small interfering (si)RNA attenuated aMPV/C-induced p53-dependent apoptosis and reactive oxygen species (ROS) generation. We also determined the effects of the PLK2 signaling pathway on aMPV/C replication, and found that inhibiting PLK2 decreased virus release at 72 h pi. Our results demonstrate that high PLK2 expression is correlated with induction of apoptosis in aMPV/C-infected Vero cells and facilitates viral release.

## 2. Materials and Methods

### 2.1. Cells and Virus Infection and Determination

Vero cells were grown at 37 °C with 5% CO2 using Dulbecco’s modified Eagle’s minimal essential medium (DMEM; Gibco, NY, USA) supplemented with 5% heat-inactivated fetal bovine serum and 1% penicillin–streptomycin solution. The avian metapneumovirus subgroup C JC strain was isolated from meat-type commercial chickens in southern China, as previously reported [11], and propagated in Vero cells. CPEs was observed under a light microscope. All the cells infected with aMPV/C followed this process. Cells were incubated with the JC strain at a multiplicity of infection (MOI) of 1 for 2 h of adsorption. After the virus was removed, the infected cells were washed in PBS and then maintained in DMEM supplemented with 2% FBS. Cells at the indicated time points were harvested and stored at −80 °C prior to analysis. Then, the viral titer was determined using a TCID_50_ assay on a monolayer of Vero cells in 96-well plates. A total of 100 μL of 10-fold serial dilutions of the supernatant samples were cultured with Vero cells in 96-well plates. At 4–5 days postinfection, CPEs were quantified and virus titer was determined as the 50% tissue culture infective dose (TCID_50_) per 0.1 mL.

### 2.2. Protein Extraction, Digestion and Labeling with iTRAQ Reagents

Vero cells at 80% confluence in 75 cm^2^ flasks were inoculated with the JC strain at an MOI of 1 for 48 and 72 h. Uninfected cells were used as controls. Cells from three independent experiments were collected with a cell scraper, washed in cold PBS and centrifuged at 500× *g* for 10 min. The proteins were extracted, and the pellets were resuspended in lysis buffer with 1 mM PMSF, 2 mM EDTA and 10 mM DTT. We then obtained solubilized protein and removed the cellular debris by sonicating and centrifuging at 4 °C and 30,000× *g*. A Bradford protein assay was used to quantify the protein concentration in the supernatants. One hundred grams of each sample was digested at 37 °C overnight with sequencing grade modified trypsin (Promega, Madison, WI, USA). Each sample was processed according to the manufacturer’s protocol for 8-plex iTRAQ reagent labeling. The labeled digested proteins were then pooled and vacuum dried.

### 2.3. SCX Chromatography and LC-MS Analysis

Strong-cation exchange (SCX) chromatography was performed with a Thermo EASY-nLC High-Performance Liquid Chromatography (HPLC) System. iTRAQ-labeled peptide mixtures were loaded onto Thermo scientific EASY columns in buffer A (10 mM KH_2_PO_4_ pH 3.0, 25% CAN). Peptides were eluted with a gradient of 0%–40% buffer B (10 mM KH_2_PO_4_ pH 3.0, 500 mM KCl, 25% ACN) for 85 min, 40%–100% buffer B for 3 min and then 100% buffer B for 2 min using a flow rate of 250 nl/min. The reconstituted samples were analyzed using a Thermo Finnigan Q Exactive Mass Spectrometer. The MS scanning conditions included 90 min in positive ion mode; 300–1800 *m*/*z*; 70,000 resolution at *m*/*z* 200; AGC target: 3e6; maximum IT: 10 ms; number of scan ranges: 1; dynamic exclusion: 40.0 s; MS2 activation type: HCD; isolation window: 2 *m*/*z*; 17,500 at *m*/*z* 200; microscans: 1; maximum IT: 60 ms; normalized collision energy: 30 eV; and underfill ratio: 0.1%.

### 2.4. Data Analysis

Relative quantification and protein identification were both performed with Proteome Discoverer 1.4 (Thermo). Trypsin was chosen due to its enzyme cleavage specificity, and there was a maximum allowed number of two missed cleavages. Carbamidomethylation and oxidation were set as fixed modifications for the iTRAQ labeling approach. The analysis was performed using a peptide tolerance of 20 ppm and a fragment tolerance of 0.6 Da using the iTRAQ method. The mass spectrometry proteomics data were deposited into the ProteomeXchange Consortium via the PRIDE [40] partner repository with the dataset identifier PXD017827.

Each MS/MS Ion search was based on the UniProt Macaca mulatta proteome database (UP000006718). The score threshold for peptide identification was set at a 1% false discovery rate (FDR). To designate significant changes in protein expression, fold changes >1.33 or <0.76 were set as cutoff values. 

We performed a GO (http://www.geneontology.org) bioinformatics analysis of DE proteins with a 1.3-fold change to catalog biological processes, cellular components and molecular functions. Significant pathway enrichment was defined using the KEGG pathway database (http://www.genome.jp/kegg/pathway.html). 

### 2.5. Knockdown of PLK2 by RNA Interference (RNAi)

Three following siRNAs were designed: siRNA-PLK2-739 (sense, 5′-CCACUACUUUGAGGACAAATT-3′; antisense, 5′-UUUGUCCUCAAAGUAGUGGTT-3′), siRNA-PLK2-1214 (sense, 5′-GCUCCUGCCAAGCACUUAATT-3′; antisense, 5′-UUAAGUGCUUGGCAGGAGCTT-3′), siRNA-PLK2-1355 (sense, 5′-CCAGAUUUCCACUUAUCAATT-3′; antisense, 5′-UUGAUAAGUGGAAAUCUGGTT-3′) and siRNA-NC (NC) (sense, 5′-UUCUCCGAACGUGUCACGUTT-3′; antisense, 5′-ACGUGACACGUUCGGAGAATT-3′). These siRNAs were designed by the GenePharma Company (Suzhou, China) and used to silence the expression of PLK2 protein in Vero cells. The monolayer cell cultures were transfected with siRNA using Lipofectamine RNAiMAX reagent according to the manufacturer’s protocol.

### 2.6. Western Blot Analysis

Western blotting was employed to analyze changes in the expression levels of proteins from the cell lysates. All protein concentrations were determined. Samples were loaded with 1 × sample loading buffer, boiled for 10 min at 100 °C, separated by SDS-PAGE and then transferred to a PE membrane. The membrane was washed and blocked for 2 h in TBST with 5% nonfat dried milk, incubated with the indicated primary antibody overnight at 4 °C and diluted in TBST according to the manufacturer’s protocol. The membrane was washed three times in TBST for 10 min and incubated for 45 min in secondary antibody diluted in PBS at room temperature. The membrane was washed three times; the target proteins were detected using a SuperSignal West Femto Substrate Trial Kit (Thermo Scientific, 34096) according to the manufacturer’s instructions and then exposed to a chemiluminescence apparatus (Proteinsample, Santa Clara, CA, USA). The following primary antibodies as following: mouse anti-F monoclonal antibodies was prepared in our laboratory; rabbit anti-PLK2 antibody (ab137539) was obtained from Abcam; mouse anti-Bcl-2 antibody (sc-7382), mouse anti-β-actin (c-4) antibody (sc-47778), mouse anti-Bax (B-9) antibody (sc-7480), mouse anti-p53 (A-1) antibody (sc-393031), mouse anti-caspase-3 (E-8) antibody (sc-7272), mouse anti-caspase-3 p11 (C-6) antibody (sc-271759), mouse anti-BIN3 (C-10) antibody (sc-514396), mouse anti-Annexin II (3D5) antibody (sc-47696) and mouse anti-Rab 8A antibody (63-BJ) (sc-81909) were purchased from Santa Cruz Biotechnology. The following secondary antibodies were obtained from Sigma-Aldrich, including horseradish peroxidase (HRP)-conjugated goat anti-mouse (A9044), anti-rabbit (A0545). 

### 2.7. Apoptosis Analysis

Vero cells were transfected with PLK2-siRNA-739 and siRNA-NC or treated with NAC (100 µM) before virus infection. After 12 h of transfection, the cells were infected with aMPV/C for 48 h. After this, the treated cells, mock-infected cells and cells infected with aMPV/C alone were collected and incubated with annexin V-fluorescein isothiocyanate (FITC) and propidium iodide (PI) for flow cytometry analysis.

### 2.8. Reactive Oxygen Species Measurement 

Vero cells were plated in 96-well plates. The cells were transfected with PLK2 siRNA-739 or siRNA- NC and 12 h later infected with aMPV/C for 48 h. After this, the treated cells, mock-infected cells and cells infected with aMPV/C alone were incubated with DCFH-DA in accordance with the manufacturer’s procedures (Beyotime Biotechnology, Shanghai, China) and analyzed by a Multiscan Spectrum. For the ROS assay, the cells were incubated with 10 µM DCFH-DA fluorescent probe for 20 min in the dark at 37 °C.

### 2.9. Measurement of Virus Release in Vero Cells

Vero cells treated with NAC or siRNA were infected with aMPV/C at MOI of 5, and cells supernatant cultures were collected at 72 hpi. The virus titer was determined as the 50% tissue culture infective dose (TCID_50_) per 0.1 mL.

## 3. Results

### 3.1. Confirmation of aMPV/C JC Propagation in Vero Cells

**Figure 1:**
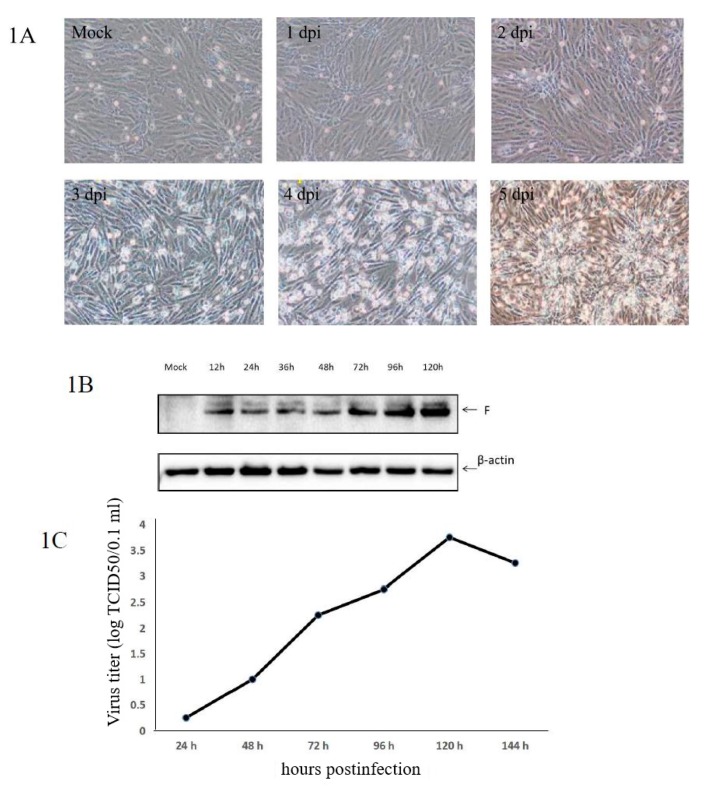
Avian metapneumovirus subtype C (aMPV/C) infection of Vero cells. (**A**) The cytopathic effects (CPEs) caused by aMPV/C JC in Vero cells at 24, 48, 72, 96, and 120 h postinfection (hpi) and mock-infected cells (control). Magnification, ×100. (**B**) Detection of aMPV/C F proteins in Vero cells after aMPV/C infection at 12, 18, 24, 48, 72, 96 and 120 hpi by Western blotting. β-Actin served as the internal control. (**C**) Growth curve of aMPV/C JC proliferation in Vero cells using the 50% tissue culture infective dose (TCID_50_) assay.

Typical cytopathic effects (CPEs) were observed in Vero cells infected with aMPV/C JC. The kinetics of aMPV/C JC propagation were determined by viral F protein expression and viral titers (50% tissue culture infective dose (TCID_50_)) at different time points post infection. As shown in Figure 1A, CPEs caused by aMPV/C JC were visible at 48 h post infection (hpi). Almost all cells became detached at 140 hpi. Western blot analysis of the aMPV/C JC infection showed that F protein was already detectable at 12 hpi (Figure 1B). The growth curve of aMPV/C JC in Vero cells showed that the viral titer increased rapidly at 48 and 72 hpi, peaked at 120 hpi and then declined (Figure 1C). A high proportion of infected cells with CPEs was regarded as optimal for proteomics. Therefore, cells at 48 and 72 hpi were chosen for further proteomic analysis.

### 3.2. Analysis of Differentially Regulated Proteins by iTRAQ-Coupled LC-MS

**Figure 2:**
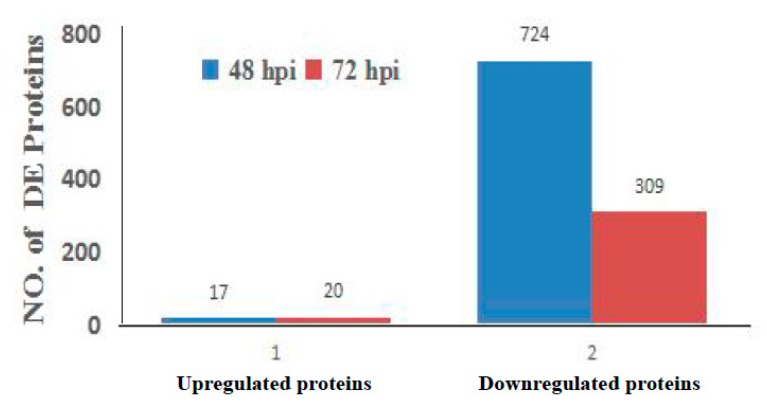
The number of significantly differentially expressed (DE) (including upregulated and downregulated) proteins in Vero cells post-aMPV/C challenge at 48 and 72 hpi with respect to mock-infected cells.

The host cell response to aMPV/C JC infection was analyzed at 48 and 72 hpi. We identified differences in protein expression between aMPV/C- and mock-infected Vero cells. All experiments were performed in triplicate. Overall, 921 of 7034 proteins were found to be significantly differentially expressed (DE; fold change (FC) >1.3 or <0.769, *p* < 0.05) using iTRAQ-coupled LC-MS/MS analysis. As shown in Figure 2, 741 and 329 proteins displayed significantly altered expression at 48 and 72 h after aMPV/C JC infection, respectively. The majority of the proteins had decreased expression in response to aMPV/C infection. Compared to that in mock-infected cells, the expression of 17 and 724 proteins was significantly up- and downregulated in infected cells at 48 hpi, respectively, while the expression of 20 and 309 proteins was significantly up- and downregulated at 72 hpi, respectively. Furthermore, the expression of 87 proteins was significantly dysregulated between the two time points.

### 3.3. Gene Ontology and KEGG Pathway Analysis

**Figure 3:**
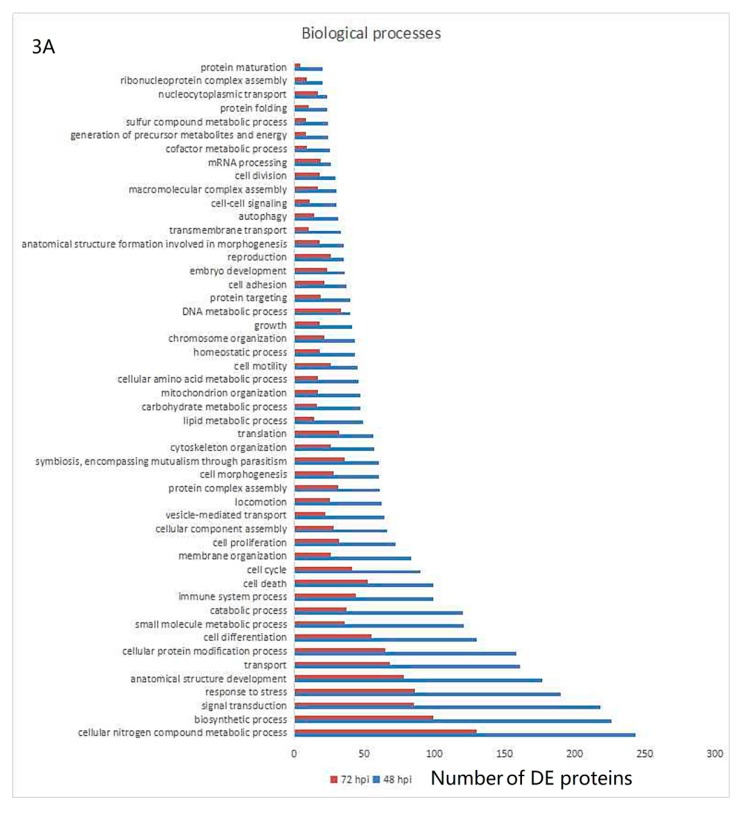

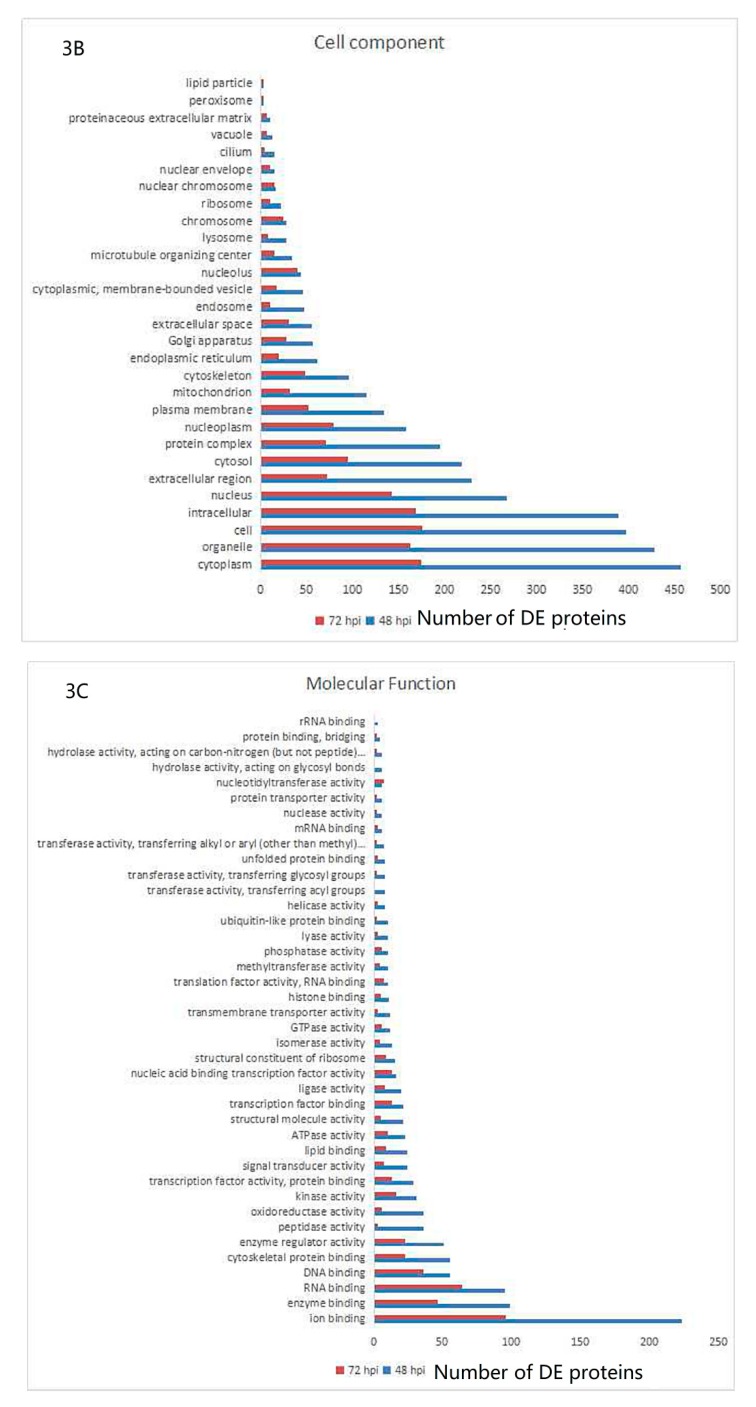
Classification of the identified DE proteins based on the gene ontology (GO) annotation. (**A**) GO biological processes. (**B**) GO cellular component. (**C**) GO molecular function. In (**A**), the most frequently represented categories were cellular nitrogen compound metabolic process, biosynthetic process and signal transduction. In (**B**), the most frequently represented categories were cytoplasm, followed by organelles and cells. In (**C**), the most frequently represented categories were ion binding, enzyme binding and RNA binding.

All DE proteins were analyzed at 48 and 72 hpi using the gene ontology (GO) database. Biological processes, cell components and molecular functions were the three main annotation types obtained from the GO analysis. The analysis of the DE proteins within the biological processes category showed that aMPV/C infection affected metabolic biosynthesis, signal transduction, host response to stress, transport, immune system process, cell death and the cell cycle (Figure 3A). These processes may be involved in infection and replication. The cellular component category revealed that the DE proteins were differentially distributed in cells (Figure 3B). The molecular function category indicated that ion binding, enzyme binding, RNA binding and DNA binding were important during aMPV/C infection (Figure 3C).

**Figure 4:**
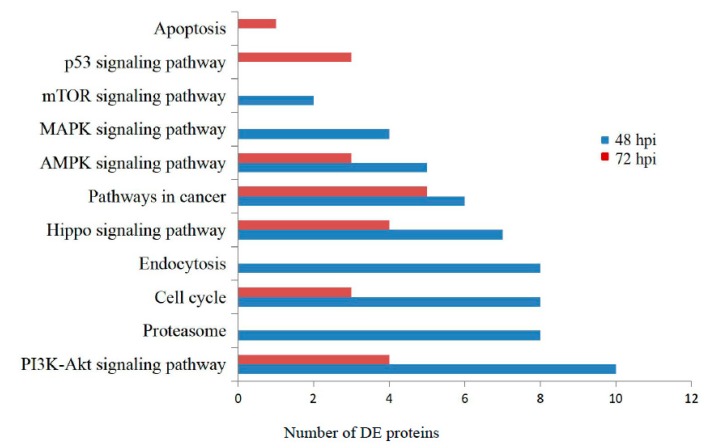
The key and representative KEGG pathway enrichment analysis of the DE proteins.

The KEGG pathway database was employed to analyze the functional classifications and signaling pathways of the identified DE proteins based on the underlying biological evidence. The significantly DE proteins induced by aMPV/C infection were clustered into several pathways, including metabolic pathways, the PI3K-Akt signaling pathway, the AMPK pathway and the cell cycle at both 48 and 72 hpi. However, for some DE proteins that are involved in endocytosis, the p53 signaling pathway and apoptosis were observed only at 48 hpi or 72 hpi (Figure 4). aMPV/C preferentially induced endocytosis and viral entry during early infection and then induced apoptosis-associated functional categories after 72 hpi.

Together, the GO and KEGG pathway analyses enabled the molecular characterization and identification of the interacting pathways associated with the DE proteins upon aMPV/C infection, which may be associated with viral infection and replication.

### 3.4. Validation of DE Proteins by Western Blotting

**Figure 5:**
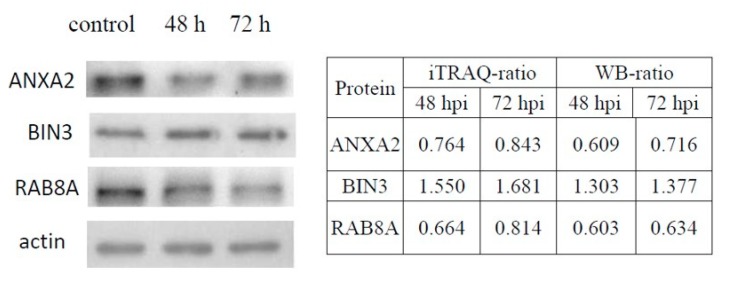
Confirmation of three DE proteins in aMPV/C-infected Vero cells by Western blot analysis. The ratios of DE proteins in iTraq and Western blots are shown in the table on the right. The relative levels of the proteins were quantified with immunoblot scanning and normalized to the amount of β-actin (B).

We examined the expression levels of BIN3 (upregulated), ANXA2 (downregulated) and RAB8A (downregulated) in Vero cells infected with aMPV/C by Western blotting. These proteins were chosen on the basis of their molecular function characteristics and their ratios. Equal amounts of cell lysates from aMPV/C-infected and control cells were tested. In Figure 5, the ratios between uninfected and infected cells at 48 and 72 hpi are shown on the right of the Western blot results. The expression changes of the three proteins were consistent with those in the proteomic analysis results using iTRAQ.

### 3.5. PLK2 Expression Is Upregulated and Involved in Apoptosis Induction in aMPV/C-Infected Cells

**Figure 6:**
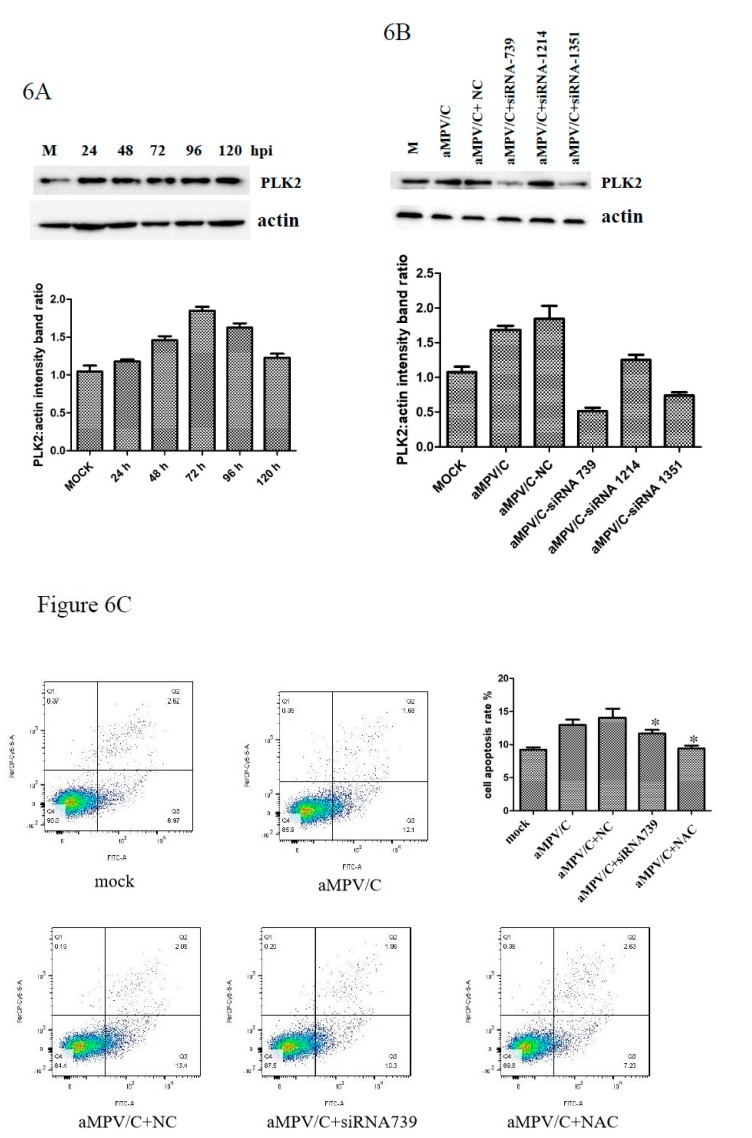
Silencing PLK2 decreases apoptosis in aMPV/C-infected cells. (**A**) PLK2 expression is upregulated during aMPV/C infection. (**B**) PLK2 expression in cells following transfection with three different siRNAs or NC (siRNA-NC) at 80 nM and subsequent infection with aMPV/C. (**C**) Apoptosis was detected by annexin V-fluorescein isothiocyanate and PI staining by flow cytometry. aMPV/C infection induced apoptosis, which was reversed by PLK2 knockdown. The cells were treated as described above. Quantification displayed with graphs representing the target protein/β-actin band ratios. Error bars: means ± SDs of three independent tests. Two-way ANOVA; * indicates a significant difference, comparing to the aMPV/C-infected group.

In the proteomics database, PLK2 expression was increased by aMPV/C infection at 48 hpi. The Western blot results confirmed this result (Figure 6A). PLK2 has been reported to be essential for cell survival regulation. We first examined whether aMPV/C infection affects apoptosis in Vero cells. The results showed significant apoptosis induction 48 h after aMPV/C infection. To investigate the function of PLK2 in aMPV/C infection, we suppressed PLK2 expression using PLK2-specific siRNAs. The results illustrated the efficiency and specificity of the PLK2 siRNAs (Figure 6B). PLK-siRNA-739 was used for further studies. N-acetylcysteine (NAC) is an antioxidant that can mitigate mitochondrial oxidative stress and apoptosis. We transfected cells with PLK-siRNA-739 or treated cells with NAC prior to infecting them with aMPV/C and detected the subsequent apoptosis. Interestingly, suppressing PLK2 partially rescued apoptosis (Figure 6C), indicating that the PLK2 protein may play an essential role in aMPV/C-induced apoptosis.

### 3.6. Silencing PLK2 Decreases aMPV/C-Induced ROS, Induction of Apoptosis, and Virus Release

**Figure 7:**
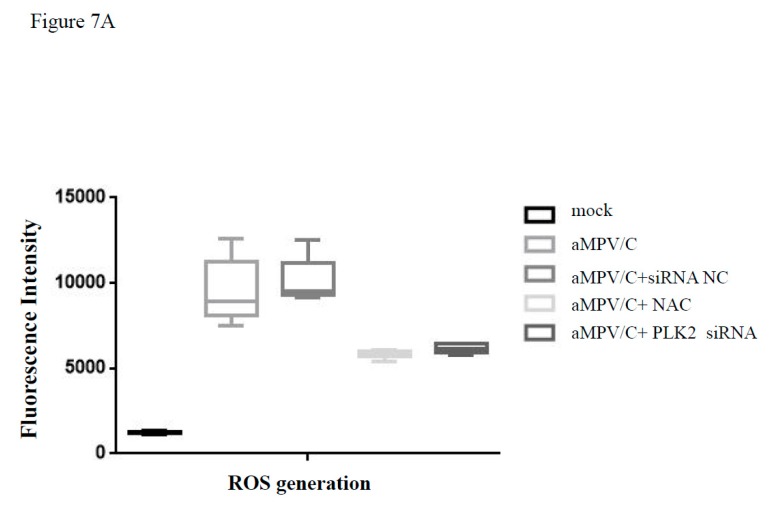

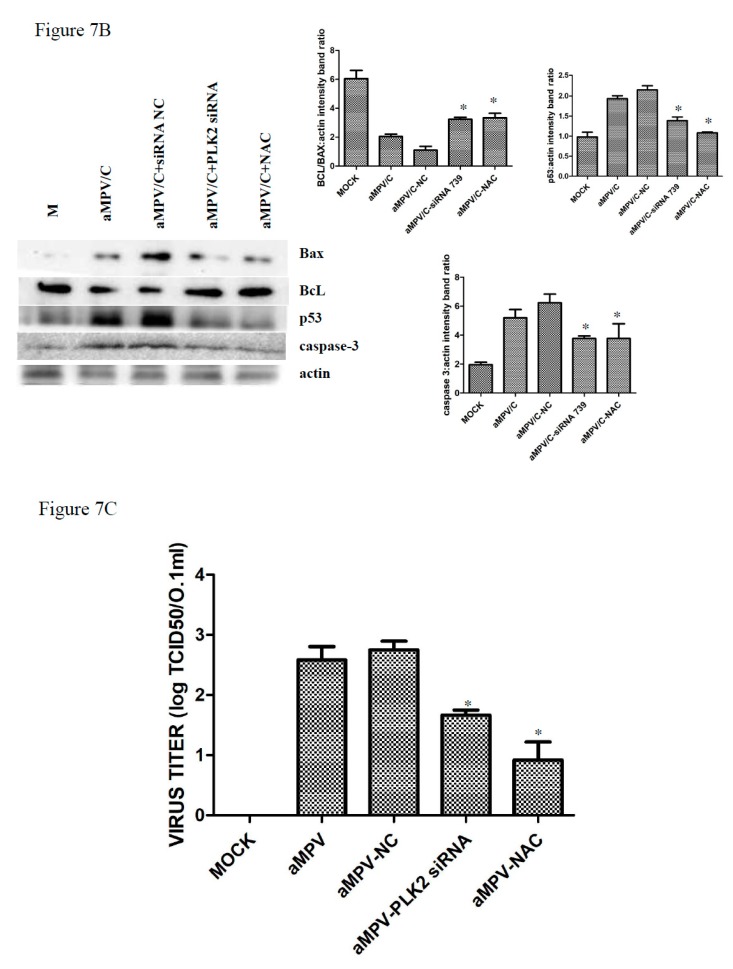
Knocking down PLK2 decreases reactive oxygen species (ROS), induction of apoptosis and virus production. (**A**) ROS was measured by a DCFH-DA fluorescent probe using Multiscan Spectrum. N-acetylcysteine (NAC) was used as a control. (**B**) Western blot analysis was used to determine the levels of apoptosis-associated proteins, p53, Bcl-2, Bax and cleaved caspase-3, in cells at 48 hpi. Quantification displayed with graphs representing the target protein/β-actin band ratios. Error bars: means ± SDs of three independent tests. Two-way ANOVA; * indicates a significant difference, comparing to the JC infected group. (**C**) The supernatant from treated and infected Vero cells was collected to determine the viral titer and compare it to that of controls using the TCID_50_ at 72 hpi. All experiments were repeated 4–6 times; * indicates a significant difference.

Apoptosis is an important component in the response to virus infection and requires specific triggering signals for activation. In addition, p53 has been intensively studied and found to mediate apoptosis. ROS can regulate p53-dependent apoptosis mediated by mitochondria. PLK2 has been reported to exert an antioxidant function and prevent p53- and ROS-coordinated cell death [39]. However, the opposite effect of PLK2 on p53 and ROS generation has also been observed in high D-glucose-induced apoptosis [41]. To investigate whether PLK2-regulated apoptosis is associated with mitochondrial dysfunction, we next measured ROS in cells after aMPV infection. Treatment with PLK2 siRNA was found to decrease ROS production in aMPV/C-infected cells (Figure 7A). However, siRNA-NC treatment did not affect ROS accumulation. We used NAC treatment as a positive control. NAC treatment blocked aMPV/C-induced apoptosis and ROS accumulation (Figure 7A). These results suggest that PLK2 contributes to aMPV/C-induced ROS production. We further studied the effect of PLK2 knockdown on apoptosis markers and found that PLK2 knockdown partially inhibited the Bcl-2/Bax ratio decrease, p53 increase and cleaved caspase-3 activation in aMPV/C-infected cells (Figure 7B). These data suggested that PLK2 induced by aMPV/C infection may contribute to apoptosis through ROS signaling.

To determine the impact of PLK2 on viral replication, we examined the virus release in cells treated with PLK2 siRNA and then infected with aMPV/C. The supernatant of infected cells in each group was collected to determine viral titers using the TCID_50_ assay. As shown in Figure 7C, compared to that in the siRNA-NC control-treated cell supernatant, aMPV/C replication in the supernatant of cells with the specific siRNA-induced knockdown of PLK2 was reduced. Taken together, these results suggest that PLK2 expression is essential for promoting aMPV/C-mediated apoptosis and increasing virus release at 72 h pi.

## 4. Discussion

Viral infections often induce host cell environment modifications, which prevent or favor viral replication. To reveal the mechanism of aMPV/C infection, we need more detailed information. Proteomics, especially iTRAQ combined with LC and tandem MS analysis, is a powerful technology used to quantitatively analyze and identify proteins and can be used to find disease-specific targets in a high-throughput manner. To date, there is no report on the cellular proteome changes after aMPV/C infection. Cultured Vero cells are the best available substrate for aMPV and have been used to study fuctions of aMPV proteins, entry to cells, mediation to membrane fusion, replication, induction of autophagy and vaccine preparation in previous reports [42,43,44,45,46,47]. aMPV/C is more closely related to hMPV and may have larger cellular host ranges potentially including mammalian than other subtypes [12,48]. In this study, iTRAQ was used for the first time to analyze Vero cells infected with aMPV/C. We identified 921 DE proteins at two time points post infection. Three selected DE proteins were confirmed using Western blotting as an independent analytical method. The proteomic results provided valuable data for the further analysis of aMPV/C infection. Bioinformatics indicated that the DE proteins are involved in a variety of processes, including metabolism, biosynthesis, signal transduction, the host response to stress, metabolic pathways, the PI3K-Akt signaling pathway, the AMPK pathway, cell death and the cell cycle (Figure 3 and Figure 4, Table 1). Our data identified several previously uncharacterized key proteins possibly involved in aMPV/C infection of Vero cells.

The cell cycle consists of DNA replication, mitosis and cytokinesis (S, M, G1 and G2 phases). Many viruses that interact with the cell cycle can subvert host-cell functions and increase viral replication. Quiescent cells are referred to as being in the G0 phase. Many host molecules participate in the regulation of the cell cycle. Cyclin-dependent kinase (CDK) 4/6 complexes and phosphorylation of the downstream retinoblastoma (Rb) protein initiate and regulate G1-phase progression. Cyclin E-Cdk2 activity is important in the G1/S transition and S phase. CDK1 is required for entry into mitosis and plays an essential role in S phase. The cell cycle is negatively regulated by cyclin-dependent kinase inhibitors (CKIs). Many viruses, including herpesviruses [49], murine hepatitis virus (MHV) [50], severe acute respiratory syndrome coronavirus (SARS-CoV) [51], influenza A virus [52], human respiratory syncytial virus [53], murine norovirus (MNV) [54] and porcine circovirus type 2 [55], have been reported to induce cell cycle arrest for efficient replication. In our study, the expression of cyclin-dependent kinase inhibitor 1B (CDN1B), cyclin-dependent kinase 6 (CDK6) and cyclin-dependent kinase 1 isoform 1 (CDK1) was found to be downregulated during aMPV/C infection in Vero cells (Table 1). These DE proteins might be responsible for cell cycle arrest during aMPV/C infection. The specific mechanism requires further investigation. 

Various cell signal transduction pathways have been shown to facilitate virus survival or the antiviral response in previous studies. The PI3K/Akt and mTOR pathways have been well documented to stimulate cell proliferation and inhibit apoptosis. Many viruses can activate the PI3K/Akt pathway to increase the viral titer. Respiratory syncytial virus (RSV), belonging to the same family as aMPV, has been shown to increase the survival of epithelial cells by activating PI3K/Akt, which increases replication before Fas-mediated apoptosis [56]. The proteins in the VTNC, 14-3-3 and RHEB pathways were found to be differentially regulated during aMPV/C infection (Table 1). The MAPK cascade pathways are part of the protein kinase cascade, which involves transcription factors and modulates cell growth, differentiation and inflammation. Some human respiratory viruses, such as RSV and H1N1 influenza virus, have been shown to utilize protein kinases, such as MAPK, for replication [57]. The expression of the STAT1, PAK2 and HSPB1 proteins was downregulated in aMPV/C-infected Vero cells (Table 1). Both up- and downregulated proteins are shown. One protein processing pathway, the heat shock response, is a multifunctional mechanism for cell survival following pathogen infection [58]. In this study, Hsp90 and Hsp70 were identified as DE proteins in Vero cells 48 h after aMPV/C infection. By regulating these pathways, the virus utilizes specific mechanisms for its infection and replication.

Apoptosis regulates the pathogenesis of many infectious diseases [59]. Apoptosis induced by virus infection can inhibit or favor viral replication. Blocking apoptosis may lead to an increased viral titer because of the increased survival of infected cells. In this study, apoptosis-related genes were identified among the DE proteins following aMPV/C infection in Vero cells. The 14-3-3 proteins represent a family of adaptor and scaffold proteins in eukaryotic organisms. They can interact with multiple cellular proteins and might be involved in modulating the cell cycle, apoptosis and cell death at multiple steps [60]. The 14-3-3 proteins have been reported to be involved in the hepatitis C virus-mediated activation of Raf-1 [61] and HIV-1-induced cell cycle regulation [62]. As shown in Table 1, the decreased expression of three 14-3-3 proteins (eta, beta/alpha, gamma) was found at 48 hpi. We speculate that the inhibition of apoptosis during early infection might contribute to the rapid replication of progeny virions before cell death. More studies are required to examine the function of the 14-3-3 proteins in aMPV/C-infected Vero cells. 

In the aMPV/C proteomic data, the proteins with decreased expression were the majority. However, we discovered significant upregulation of PLK2 expression in aMPV/C-infected cells, which was further confirmed by Western blotting. Polo-like kinases are related to serine/threonine protein kinases, which are well-defined cell cycle regulators. PLK2 has been reported to act as a tumor suppressor gene but sometimes plays an oncogenic role in tumors. PLK2 can affect cell cycle regulation and cell survival, which is closely associated with the p53 family and ROS signaling pathway. We found that aMPV/C infection triggers apoptosis and ROS generation in Vero cells, which is inhibited by PLK2 siRNA. Considering that the p53 signaling pathway is involved in aMPV/C infection, the measurement of Bcl-2, Bax, p53 and caspase-3 was performed in aMPV/C-infected Vero cells. The BCL-2 family is a key regulator of mitochondrial apoptosis that comprises proapoptotic and antiapoptotic members. BCL-2 belongs to antiapoptotic proteins. Bax is a proapoptotic effector protein. Specific cellular signaling can modulate the expression of these two kinds of proteins to promote cell survival or death. A family of cysteine proteases called the caspase cascade can mediate the process of apoptosis. Caspase-3 is the final common molecule involved in apoptosis. A decreased Bcl-2/Bax ratio can damage the integrity of mitochondria and lead to caspase-3 activation during apoptosis. In our report, suppressing PLK2 inhibited ROS production and reversed the apoptosis marker expression changes, including an increased Bcl-2/Bax ratio and decreased cleaved caspase-3 and p53 expression. In the current study, our data collectively suggest for the first time that PLK2 expression contributes to p53-mediated apoptosis and ROS generation in aMPV/C-infected Vero cells. This result demonstrates that PLK2 plays an important role in aMPV/C-induced apoptosis through ROS production and activation of the p53 signaling pathway. To a certain extent, this result is consistent with those of previous reports showing the proapoptotic function of PLK2 but not its antioxidant function [39]. Through further investigation, we demonstrated that the inhibition of PLK2 expression decreases virus production in the supernatant at 72 h pi. We speculate that PLK2 might promote apoptosis for viral release. The findings of this study suggest that PLK2 can regulate p53 and ROS and plays an essential role in apoptosis and viral replication in Vero cells after aMPV/C infection.

aMPV/C infection has recently emerged in China and has been gaining increasing attention due to its high morbidity rates and pathogenicity. In general, this is the first report of the proteomic profiles of Vero cells following aMPV/C infection using iTRAQ combined with LC-MS. These data provide us comprehensive information about several DE proteins in cellular responses regarding cell cycle, endocytosis, and PI3K-Akt, mTOR, MAPK and p53 signaling pathway in the cultured aMPV/C-infected Vero cells. Furthermore, a high expression of PLK2 by aMPV/C infection promoted apoptosis through ROS accumulation and p53 signaling activation, and release of the virus. However, in-depth analysis of host cellular responses to aMPV/C infection in natural host-chickens will be needed to better understand the pathogenic mechanisms for the development of vaccine strategies and antiviral therapeutic targets for aMPV/C infection in the future.

## Figures and Tables

**Table 1:** Some statistically significant DE proteins identified by iTRAQ analysis of Vero cells infected with aMPV/C. (PI3K-AKT pathway/cell cycle/MAPK/endocytosis) (ratio >1.3 or < 0.769).

Protein Name	Accession No.	Ratios (Infection/Control)		Peptides	Sequence Coverage
		48 hpi	72 hpi		
14-3-3 protein beta/alpha	F6XFG9	0.726 #	0.792	16	70.33
Cyclin-dependent kinase inhibitor 1B	F6Z4R0	0.732 #	0.922	2	10.61
Somatomedin-B	VTNC	1.418	1.432	2	4.8
14-3-3 protein eta	F6RIX6	0.700#	0.799	17	72.16
14-3-3 protein gamma	F6XLR8	0.689#	0.778	18	85.02
GTP-binding protein Rheb	F7H5C5	0.694#	0.751	5	27.72
Cyclin-dependent kinase 6	F7E5V8	0.753#	0.791	12	42.94
Heat shock protein beta-1	F6XTF7	0.759#	0.818	16	84.88
Non-specific serine/threonine protein kinase	F7GWI2	0.766#	0.765#	17	43.51
Signal transducer and activator of transcription	F6WE02	0.651#	0.732#	24	38.67
Cyclin-dependent kinase 1 isoform 1	F7GRY6	0.745#	0.738#	13	53.2
